# The role of risk factors in development of dementia in the Czech Republic

**DOI:** 10.1002/alz.092375

**Published:** 2025-01-09

**Authors:** Matěj Kučera

**Affiliations:** ^1^ Vrije Universiteit Amsterdam, Amsterdam, Netherlands; 2nd Medical School, Charles University, Prague, Czech Republic; The National Institute of Mental Health, Klecany Czech Republic

## Abstract

**Background:**

In 2020, the Lancet Commission introduced a meta‐analysis featuring a life course model encompassing 12 potentially modifiable risk factors for dementia: low education, hypertension, hearing impairment, smoking, obesity, depression, physical inactivity, diabetes, low social contact, alcohol consumption, traumatic brain injury, and air pollution. The aim of this study is to investigate the association of the aforementioned risk factors with the incidence of dementia with the incidence of dementia in the Czech Republic, capitalizing on a nation‐wide population‐based study.

**Method:**

The analysis was based on data from Survey on Health, Ageing and Retirement in Europe (SHARE). Participants were included in the analytical sample if they fulfilled the following criteria: age of entry to the study of 65 years and higher, participation in at least two waves of SHARE, at least two measurements of cognitive function, and dementia‐free state at baseline. To study the association of risk factors with the incident dementia, we utilized Cox hazards models estimating hazard ratio (HR) with 95% confidence interval (CI).

**Result:**

Out of the ten risk factors, five presented an association with dementia, when adjusted for age, sex and birth cohort. The strongest hazard was found for physical inactivity, followed by low education, diabetes mellitus, depression and living alone. There was a tendency of sex being an effect modifier in the association of alcohol use, living alone and low education with incident dementia.

**Conclusion:**

In the present study, capitalizing on more than 3,500 community‐dwelling older adults, we found an association for only half of the proposed risk factors. More systematic attention should be paid to mitigating the negative effect of dementia risk factors as this could contribute to the reduction of incident dementia cases in the Czech Republic, Future studies should replicate the findings of this study in other CEE countries and ideally should have a longer follow‐up period to prevent reverse causation.

